# Conformational Plasticity and Ligand Binding of Bacterial Monoacylglycerol Lipase*

**DOI:** 10.1074/jbc.M113.491415

**Published:** 2013-09-06

**Authors:** Srinivasan Rengachari, Philipp Aschauer, Matthias Schittmayer, Nicole Mayer, Karl Gruber, Rolf Breinbauer, Ruth Birner-Gruenberger, Ingrid Dreveny, Monika Oberer

**Affiliations:** From the ‡ Institute of Molecular Biosciences, University of Graz, Humboldtstrasse 50/3, A-8010 Graz, Austria,; the § Institute of Pathology and Centre of Medical Research, Medical University of Graz, A-8010 Graz, Austria,; the ¶ Institute of Organic Chemistry, Graz University of Technology, Stremayrgasse 9, A-8010 Graz, Austria, and; the ‖ Centre for Biomolecular Sciences, School of Pharmacy, University of Nottingham, Nottingham NG7 2RD, United Kingdom

**Keywords:** Crystal Structure, Lipase, Lipids, Lipid Metabolism, Monoacylglycerol, Monoglyceride, X-ray Crystallography, MGL, Phosphonate Inhibitor, Substrate Analogs

## Abstract

Monoacylglycerol lipases (MGLs) play an important role in lipid catabolism across all kingdoms of life by catalyzing the release of free fatty acids from monoacylglycerols. The three-dimensional structures of human and a bacterial MGL were determined only recently as the first members of this lipase family. In addition to the α/β-hydrolase core, they showed unexpected structural similarities even in the cap region. Nevertheless, the structural basis for substrate binding and conformational changes of MGLs is poorly understood. Here, we present a comprehensive study of five crystal structures of MGL from *Bacillus* sp. H257 in its free form and in complex with different substrate analogs and the natural substrate 1-lauroylglycerol. The occurrence of different conformations reveals a high degree of conformational plasticity of the cap region. We identify a specific residue, Ile-145, that might act as a gatekeeper restricting access to the binding site. Site-directed mutagenesis of Ile-145 leads to significantly reduced hydrolase activity. Bacterial MGLs in complex with 1-lauroylglycerol, myristoyl, palmitoyl, and stearoyl substrate analogs enable identification of the binding sites for the alkyl chain and the glycerol moiety of the natural ligand. They also provide snapshots of the hydrolytic reaction of a bacterial MGL at different stages. The alkyl chains are buried in a hydrophobic tunnel in an extended conformation. Binding of the glycerol moiety is mediated via Glu-156 and water molecules. Analysis of the structural features responsible for cap plasticity and the binding modes of the ligands suggests conservation of these features also in human MGL.

## Teaser

**Background:** Monoacylglycerol lipases hydrolyze monoacylglycerols into free fatty acids and glycerols.

**Results:** Crystal structures provide the structural basis for conformational plasticity and ligand binding of a monoacylglycerol lipase.

**Conclusion:** Hallmark features responsible for substrate binding and selectivity are conserved across species.

**Significance:** The first structures of a monoacylglycerol lipase in complex with substrate analogs are presented.

## Introduction

Understanding the molecular basis of enzyme substrate interactions is an important prerequisite for elucidating substrate structure-activity relationships. Structures of protein·ligand complexes are critical in delineating ligand interactions with the binding site, uncovering active site residues, and providing insight into the catalytic mechanism (1). In addition, they also provide valuable information on the “druggability” of the binding site, laying the basis for structure-based drug design (1, 2).

Lipases catalyze the hydrolysis of ester bonds in long chain acylglycerols releasing fatty acids and glycerol. Most lipases possess a lid or cap domain that covers the active site and is able to undergo conformational changes to carry out catalysis at the lipid-water interface (3, 4). The nature of these conformational changes is diverse across the lipase family and is poorly understood at an atomic level. In this study, we provide in-depth experimental insight into conformational changes and substrate binding observed in monoacylglyerol lipases (MGLs).^4^ MGLs are a unique class of lipases (EC 3.1.1.23) because they specifically cleave monoacylglycerols (MGs) in contrast to other lipases, *e.g.* hormone-sensitive lipase, which accepts a broad range of substrates (triacylglycerol, diacylglycerol, cholesterol esters, and MGs), and other fungal lipases (5–8). MG-hydrolyzing lipases were first described in the 1960s, and several orthologs of MGL have been characterized over the years (8–13). The physiological function of MGL is best understood in mammals, where it has an essential role in lipid metabolism for maintaining energy homeostasis (14). Additionally, MGL plays an important part in mediating endocannabinoid-based signaling rendering it an important pharmacological target (15–17). In bacteria, MGLs are thought to have a role in detoxification processes because short chain MGs are highly toxic to these organisms (18–20).

Crystallographic studies of MGLs have resulted in the determination of three-dimensional structures of human MGL (hMGL) and its ortholog from a moderately thermophilic soil bacterium *Bacillus* sp. H-257 (bMGL) in free form and in complex with inhibitors (21–24). Both MGLs possess the expected α/β-hydrolase core domain harboring a catalytic triad. Additionally, the structures revealed an unexpected conservation of the overall cap architecture between hMGL and bMGL (24). Thus, bMGL can be used as an excellent model system to study the mode of action of MGLs and relate these insights to eukaryotic MGLs.

Interestingly, the cap domain of hMGL was reported to adopt open and closed conformations, whereas only an open conformation was observed for bMGL. Very little is known about the effect of these conformational changes on enzyme activity (23, 24). Similarly, the structural basis for the different substrate turnover rates shown by bMGL is also poorly understood. Reportedly, bMGL possesses higher turnover rates for MGs with shorter chain length, *i.e.* C_10:0_ and C_12:0_, compared with longer chain MGs, *i.e.* C_18:0_ and C_18:1_ (25, 26).

In this work, we describe five crystal structures of bMGL in its free form and in complex with substrate analogs and the natural substrate 1-lauroylglycerol (1-LG). We discuss their structure-function relationship, enzymatic assays, and mutations of the bacterial MGL from *Bacillus* sp. H-257. The bMGL·ligand complexes reported here represent the first experimental structures of substrate or its analogs bound to an MGL. These complex structures provide experimental evidence for conformational plasticity in the cap region of bMGL. Additionally, these structures also elucidate the molecular basis of substrate binding and help to rationalize differences in substrate turnover rates.

## EXPERIMENTAL PROCEDURES

### Cloning, Expression, and Purification of bMGL Mutants

Site-directed mutagenesis (I145G, I145S, and D196N) was employed to introduce single point mutations in the bMGL gene. Primers (Invitrogen) used were as follows: I145G, forward, GCCGAGGTATCTGGATTCGGGCGGTTCGGACTTG, and reverse, CAAGTCCGAACCGCCCGAATCCAGATACCTCGGC; I145S, forward, GCCGAGGTATCTGGATTCGAGCGGTTCGGACTTG, and reverse, CAAGTCCGAACCGCTCGAATCCAGATACCTCGGC; and D196N, forward, TTTTGTCTCCGACGAAAATCACGTCGTGCCGC, and reverse, GCGGCACGACGTGATTTTCGTCGGAGACAAAA. PCRs were performed using standard protocols. After DpnI digestion, PCR products were transformed into TOP10 chemically competent *Escherichia coli* (Invitrogen). Mutations were verified by DNA sequencing, and variant enzymes were expressed and purified as described previously (24).

### Synthesis of Substrate Analogs

**FIGURE 1.**
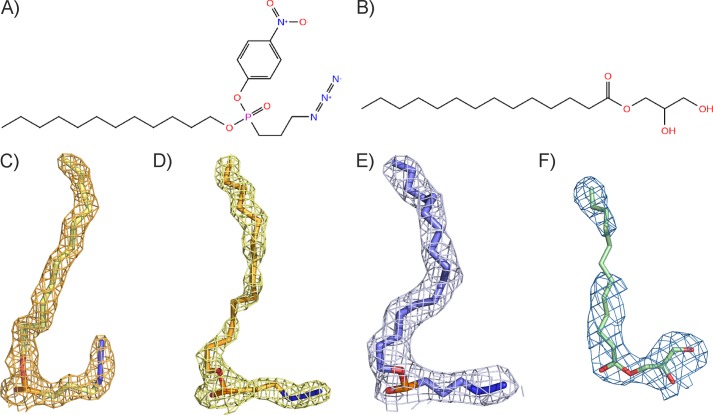
**Substrate and substrate analogs observed in the bMGL complex structures.** Structural formula of the C_12_ ligand (*A*) mimicking 1-myristoylglycerol (C_14:0_) (*B*). *C–E*, 2*F_o_* − F*_c_* maps (*mesh*) of the C_12_ ligand (*yellow sticks*), C_14_ ligand (*orange sticks*), and the C_16_ ligand (*slate blue sticks*). *F*, 2*F_o_* − F*_c_* (*blue mesh*) of 1-LG (*green sticks*) bound to bMGL contoured at 1σ level.

The synthesis of the ligands with different alkyl chain lengths was carried out under an inert atmosphere. Two equivalents of sodium azide (1.15 g, 17.8 mmol) were added to a solution of diethyl-(3-bromopropyl)-phosphonate (2.30 g, 8.88 mmol) in 20 ml of THF/H_2_O (1:1), and the reaction mixture was heated for 5 h at 80 °C. After cooling to room temperature, the pH was adjusted to 10 by addition of solid NaOH. Extraction with Et_2_O (three times, 20 ml), drying over MgSO_4_, and concentrating *in vacuo* furnished diethyl-(3-azidopropyl)-phosphonate in quantitative yield. 500 mg (2.26 mmol) of the resulting crude product were dissolved in 2.1 ml of CH_2_Cl_2_ in a Schlenk tube. Trimethylsilyl bromide (0.92 ml, 7.0 mmol) was added, and the reaction was stirred at room temperature for 5 h. After concentration *in vacuo* (using a liquid nitrogen trap), crude bis(trimethylsilyl)-(3-azidopropyl)-phosphonate was produced. The yellow oil was again dissolved in 3 ml of CH_2_Cl_2_ in the same Schlenk tube. Oxalylchloride (4 eq) and a catalytic amount of *N,N*-dimethylformamide were added dropwise, and the reaction mixture was stirred at 40 °C for 75 min. After removal of all volatiles *in vacuo* (using a liquid nitrogen trap) crude dichloro-(3-azidopropyl)-phosphonate remained in the Schlenk tube. In an extra Schlenk tube, a solution of 2.26 mmol of alcohol (of different chain lengths), 0.46 ml of *N*,*N*-diisopropylethylamine, 0.41 ml of tetrazole, and 0.34 ml of diazabicyclo-[5.4.0]undec-7-ene in 2.1 ml of THF was prepared and then added in 0.5-ml portions to the other Schlenk tube containing the substrate. After overnight stirring, the resulting suspension, a solution of 4-nitrophenol (470.8 mg, 3.38 mmol) and *N*,*N*-diisopropylethylamine (0.92 ml) in 2.1 ml of THF (which had been stirred overnight as well), was added. After stirring for 2 h at room temperature, the reaction mixture was concentrated using a rotary evaporator. The crude product was dissolved in CH_2_Cl_2_ (30 ml) and washed with H_2_O (two times, 15 ml). After back-extraction with CH_2_Cl_2_ (20 ml), the combined organic phases were washed with brine (25 ml), dried over MgSO_4_, concentrated, and dried in an oil pump vacuum yielding the desired alkyl-4-nitrophenyl (3-azidopropyl)phosphonate product (*e.g.* dodecyl 4-nitrophenyl (3-azidopropyl)phosphonate). The dried crude product was purified by preparative HPLC. It should be noted that a substrate analog with a 12-carbon alkyl chain actually corresponds to monomyristoylglycerol (C_14:0_) rather than monolauroylglycerol (C_12:0_) (Fig. 1).

#### 

##### Crystallization of Substrate Analog Complexes

For the bMGL·C_12_ ligand complex, a 0.9 mm solution of bMGL was mixed with 50 mg/ml of the C_12_ ligand dissolved in DMSO (Merck) to achieve a final concentration of 4.5 mm of the ligand. Crystallization trials were performed using the sitting drop vapor diffusion method with equal volumes of the bMGL·C_12_ complex and the Morpheus screen D4 (Molecular Dimensions, Suffolk, UK) reservoir solution containing 0.1 m MES/imidazole, pH 6.5, 12.5% w/v PEG 1000, 12.5% w/v PEG 3350, 12.5% v/v 2-methyl-2,4-pentanediol, and 0.02 m alcohols (0.2 m 1,6-hexanediol, 0.2 m 1-butanol, 0.2 m (*RS*)-1,2-propanediol, 0.2 m 2-propanol, 0.2 m 1,4-butanediol, 0.2 m 1,3-propanediol). Well diffracting crystals (1.7 Å) were obtained from the drop without further optimization within 2 weeks, and no additional cryoprotectant was used for flash cooling the crystals in liquid nitrogen. For the bMGL·C_14_ ligand complex, a 0.9 mm solution of bMGL was mixed with 50 mg/ml C_14_ ligand, dissolved in 99% EtOH (Sigma) to achieve a final ligand concentration of 4.5 mm, and incubated for 1 h at 4 °C. Well diffracting crystals (1.8 Å) were obtained after 2 weeks using the hanging drop method in a crystallization condition containing 0.1 m citric acid, pH 5.2, and 18% PEG 3350. For the bMGL·C_16_ ligand complex, a 0.9 mm solution of bMGL was mixed with 50 mg/ml C_16_ ligand, dissolved in 99% EtOH (Sigma) to achieve a final ligand concentration of 4.5 mm, and incubated for 1 h at 4 °C. Crystals diffracting to 2.2 Å were obtained in ∼10 days from the drop containing 0.1 m citric acid, pH 5.0, and 22% PEG 3350 using the hanging drop method.

##### Crystallization of the bMGL(D196N) Variant in Complex with Substrates

An ∼0.9 mm solution of bMGL(D196N) was mixed with 180 mm 1-LG (Sigma) dissolved in 99% EtOH to achieve a final protein/ligand ratio of 1:5. The protein/ligand mixture was incubated at 4 °C for 1 h. Initial crystals were obtained using the Morpheus screen, condition 4, containing 0.03 m magnesium chloride, 0.03 m calcium chloride, 0.1 m MES/imidazole, pH 6.5, 12.5% 2-methyl-2,4-pentanediol, 12.5% PEG 1000, and 12.5% PEG 3350. These crystals were used for preparing a seed stock. The optimized crystals were obtained in a drop containing 0.9 mm bMGL(D196N), 56% v/v MPD, 0.1 m HEPES, pH 6.9, and 1:1000 dilution of seed stock in a ratio of 2:2:1 respectively. To increase the probability of 1-LG being bound in the structure, we also added 1-LG as powder to these crystallization drops and soaked the crystals for 8 h. Approximately 0.9 mm bMGL(D196N) was also crystallized in the presence of 140 mm 1-(*rac*)-oleoylglycerol (1-OG). Crystals diffracting to 1.7 Å were obtained from the drop containing 54% v/v MPD, 0.1 m HEPES, pH 6.9, and 1:1000 dilution of seeding stock in a ratio of 2:2:1, respectively.

### Data Processing and Structure Refinement

Datasets were recorded at beamlines indicated in Tables 1–3. All structures were solved using molecular replacement with the structure of bMGL in its free form (PDB code 3RM3) as search template (24). Models of the ligands were created with the program MAESTRO (Maestro, version 9.3, Schrödinger, LLC, New York) and were used as input for PHENIX.elbow for creating Crystallographic Information File (CIF) library files containing bond length and angle restraints (27). The models were then subjected to rigid body and restrained refinement cycles using the program REFMAC5, followed by several iterative rounds of refinement using PHENIX (28, 29). There, water molecules were added, and the weights for the x-ray/stereochemistry and x-ray/ADP were optimized resulting in the lowest *R*_free_ value. COOT was used to manually adjust and monitor the structures and the solvent molecules (30).

**TABLE 1:** **Data collection and refinement statistics** To calculate *R*_free_, 5% of the reflections were excluded from the refinement. *R*_sym_ is defined as *R*_sym_ = Σ*_hkl_*Σ*_i_*|*Ii*(*hkl*)−〈*I*(*hkl*)〉|/Σ*_hkl_*Σ*_i_Ii*(*hkl*). Data in parentheses correspond to the highest resolution shell. r.m.s.d., root mean square deviation.

Contents	bMGL·C_12_ complex	bMGL·C_14_ complex
**Data collection**		
Beamline	X13-DESY	SLS:PXIII-X06DA
Wavelength	0.81 Å	1.0 Å
Resolution	25.0 to 1.7 Å	43.7 to 1.85 Å
Space group	*P*2_1_	*P*2_1_
Unit cell parameters		
*a, b, c*	43.8, 71.2, 72.9 Å	77.0, 81.1, 85.6 Å
β	102.0°	100.3°
Total no. of reflections	143,465	293,651
Unique reflections	47,227	88,070
*R*_sym_	0.065	0.041 (0.15)
Completeness	96.9% (87.8%)	99.5% (99.6%)
Mean *I*/σ(*I*)	8.7 (4.2)	17.9 (4.2)
Multiplicity	3.0 (2.8.)	3.4 (3.3)

**Refinement statistics**		
No. of protein atoms	3853	7668
No. of solvent molecules	439	1200
*R*_work_	17.3	18.0
*R*_free_	20.8	22.0

**Model geometry**		
r.m.s.d. bonds	0.007 Å	0.007 Å
r.m.s.d. angles	1.109°	1.043°

**Ramachandran distribution**		
Most favored	97.5%	97.3%
Additionally allowed	2.5%	2.7%
Outliers	0.0%	0.0%

**TABLE 2:** **Data collection and refinement statistics** To calculate *R*_free_, 5% of the reflections were excluded from the refinement. *R*_sym_ is defined as *R*_sym_ = Σ*_hkl_*Σ*_i_*|*Ii*(*hkl*)−〈*I*(*hkl*)〉|/Σ*_hkl_*Σ*_i_Ii*(*hkl*). Data in parentheses correspond to the highest resolution shells. r.m.s.d., root mean square deviation.

Contents	bMGL·C_16_ complex
**Data collection**	
Beamline	ESRF ID 14–4
Wavelength	0.98 Å
Resolution	43.7 to 2.2 Å
Space group	*P*2_1_
Unit cell parameters	
*a, b, c*	76.9 80.3 85.7 Å
β	100.02°
Total no. of reflections	339,325
Unique reflections	48,702
*R*_sym_	0.14 (0.56)
Completeness	93.2% (68.4%)
Mean *I*/σ(*I*)	9.0 (4.3)
Multiplicity	7.0 (4.8)

**Refinement statistics**	
No. of protein atoms	7384
No. of solvent molecules	227
*R*_work_	22.3%
*R*_free_	26.2%

**Model geometry**	
r.m.s.d. bonds	0.003 Å
r.m.s.d. angles	0.840°

**Ramachandran distribution**	
Most favored	96.2%
Additionally allowed	3.8%
Outliers	0.0%

**TABLE 3:** **Data collection and refinement statistics** To calculate *R*_free_, 5% of the reflections were excluded from the refinement. *R*_sym_ is defined as *R*_sym_ = Σ*_hkl_*Σ*_i_*|*Ii*(*hkl*) − 〈*I*(*hkl*)〉|/Σ*_hkl_*Σ*_i_Ii*(*hkl*). Data in parentheses correspond to the highest resolution shell. r.m.s.d., root mean square deviation.

Contents	bMGL·1-LG complex	bMGL free form in *P*2_1_2_1_2_1_
**Data collection**		
Beamline		SLS:PXIII-X06DA
Wavelength	1.54 Å	1.0 Å
Resolution	73.62 to 2.8 Å	19.9 to 1.7 Å
Space group	*P*2_1_2_1_2_1_	*P*2_1_2_1_2_1_
Unit cell parameters *a, b, c*	39.19, 182.88, 248.26 Å	39.17, 183.13, 244.70 Å
Total no. of reflections	166,183	159,4737
Unique reflections	42,482	195,079
*R*_sym_	0.096 (0.401)	0.051 (0.406)
Completeness	94.4% (87.6%)	99.99% (100%)
Mean *I*/σ(*I*)	9.4 (2.6)	22.6 (4.4)
Multiplicity	3.9 (3.1)	8.2 (6.6)

**Refinement statistics**		
No. of protein atoms	11,010	115,60
No. of solvent molecules	39	934
*R*_work_	20.0%	19.1%
*R*_free_	24.5%	21.1%

**Model geometry**		
r.m.s.d. bonds	0.008 Å	0.006 Å
r.m.s.d. angles	1.360°	0.954°

**Ramachandran distribution**		
Most favored	95.91%	96.2%
Additionally allowed	3.87%	3.5%
Outliers	0.22%	0.3%

Differences in processing of datasets, *R*-values, and Ramachandran plot analysis after validation of the models using the MolProbity server are listed separately below (31). All figures displaying structures were generated using PyMOL (32).

#### 

##### bMGL·Ligand Complexes

The bMGL·C_12_ ligand complex dataset (1.7 Å) was indexed and integrated using iMosflm and scaled using Scala (33, 34). Molecular replacement was carried out using the program Phaser (35). *B* factors of the atoms were refined anisotropically. The final model has an *R*_work_ of 17.3% and an *R*_free_ of 20.8%. All amino acids were in the allowed regions of the Ramachandran plot. In the final model (PDB code 4KE7), no electron density was observed for residues Thr-133–Glu-137. The bMGL·C_14_ ligand complex dataset (1.85 Å) was indexed and integrated using XDS and scaled using Scala (34, 36). Molecular replacement was carried out using the Balbes server (37). Model building was performed using Arp/wArp (38). The final model (PDB code 4KE8) has an *R*_work_ of 17.8% and an *R*_free_ of 21.8%. All amino acids were in the allowed regions of the Ramachandran plot. X-ray diffraction data of the bMGL·C_16_ ligand complex (2.2 Å) were indexed and integrated using XDS and scaled using Scala (34, 36). Rigid body refinement was carried out with PHENIX (29). The Ramachandran plot indicated 100% of the residues in the allowed regions. The final model (PDB code 4KE9) has an *R*_work_ of 22.3% and an *R*_free_ of 26.2%.

##### bMGL(D196N) Structures

For the bMGL(D196N)·1-LG complex structure, diffraction data were collected to 2.8 Å on a Schneider x-ray generator equipped with a Mar345 Imaging plate detector (University of Graz, Institute for Molecular Biosciences). This dataset was indexed and integrated using iMosflm and scaled using Scala (33, 34). Two datasets (1.7 and 3.14 Å) were collected for crystals of bMGL(D196N) that had been crystallized in the presence of 1-OG. The datasets were indexed and integrated using XDS and merged in Pointless and scaled using Scala. Initial rigid body refinement was performed using Refmac5, and further refinement steps were carried out using PHENIX (29). COOT was used to manually adjust and monitor the structure and solvent molecules (30). The bMGL(D196N)·1-LG complex structure (PDB code 4KE6) was refined to final values of *R*_work_ = 20.0% and *R*_free_ = 24.5%, respectively. The structure of bMGL(D196N) crystallized in the presence of 1-OG (PDB code 4KEA) was refined to final values of *R*_work_ = 19.1% and *R*_free_ = 21.1%, respectively.

### Monoacylglycerol Hydrolase Activity Assay

Monoacylglycerol hydrolase activity of bMGL was assayed similarly to a protocol described previously (13). The assays were performed to compare the activity of wild-type (WT) bMGL with bMGL-I145G and bMGL-I145S variants. Solutions of 68 nm WT bMGL, 82 nm I145G mutant, and 96 nm I145S mutant in 10 μl were incubated with 100 μl of substrate containing either 1 mm 1-OG or 1-LG (Sigma) and complexed to defatted BSA in 100 mm potassium phosphate buffer, pH 7.4. Reactions were carried out at 37 °C for 10 min, which is in the linear range of the reaction. The reaction was stopped by the addition of 100 μl of chloroform; samples were centrifuged at 16,100 × *g* for 10 min, and 50-μl aliquots of the upper phase were collected to determine the free glycerol concentration using a commercial kit (Sigma). Assays were performed in triplicate in at least three independent experiments.

#### 

##### Protein Data Bank Accession Numbers

Coordinates and structure factors have been deposited in the Protein Data Bank under accession codes 4KE6 (bMGL(D196N)·1-LG complex), 4KE7 (bMGL·C_12_ ligand complex), 4KE8 (bMGL·C_14_ ligand complex), 4KE9 (bMGL·C_16_ ligand complex), and 4KEA (uncomplexed bMGL(D196N)).

## RESULTS AND DISCUSSION

The physiological roles of MGL in hydrolyzing MG has been known for decades, yet three-dimensional structural analyses of MGLs that provide a rationale for understanding the substrate selectivity are limited at present (5, 6, 21–23). Structural studies of human and bacterial MGLs unveiled an unexpected, yet striking, similarity in the overall architecture of the cap region. Open and closed conformations of the cap region have been observed in human MGL in its free form and in the presence of different inhibitors (21–23). The three-dimensional structures of bMGL in its free form and in complex with the irreversible inhibitor PMSF also represent a snapshot of the lipase in an open conformation (24). Molecular dynamics simulation of bMGL suggested conformational plasticity in the cap region, also suggesting the existence of closed conformations that restrict access to the active site (24).

For this work, we wanted to capture different conformations of bMGL and elucidate the exact binding site of bMGL for its MG substrates. Therefore, we determined crystal structures of bMGL in complex with ligands of different alkyl chain lengths (12, 14, and 16 carbons; subsequently referred to as C*_x_* ligands, whereby *x* refers to the number of carbons in the alkyl chain) to mimic the MG substrates myristoyl-, palmitoyl-, and stearoylglycerol (Fig. 1, *A* and *B*). These structures provide the experimental evidence for conformational plasticity of the cap region in bMGL and provide insights into the first steps of the hydrolytic reaction at atomic detail. Additionally, the three-dimensional structures of the bMGL·C_12_, bMGL·C_14_, and bMGL·C_16_ ligand complexes reveal the molecular basis of ligand binding of the alkyl chain moiety of the MG substrate. Furthermore, we generated an inactive bMGL variant, soaked its crystals with 1-LG, and examined interactions of the glycerol headgroup of this natural substrate with bMGL.

### Structure Determination of Substrate and Substrate Analog Complexes

bMGL was co-crystallized with the C_12_ ligand mimicking a C_14_ alkyl MG yielding crystals of space group *P*2_1_, which diffracted to a resolution of 1.7 Å. Two molecules of bMGL were in the asymmetric unit, and both had the C_12_ ligand covalently bound in the active site; clear electron density for the entire ligand was observed in both chains (Fig. 1*C*). No electron density was observed for residues Thr-133–Glu-137 indicating their flexibility (PDB code 4KE7).

The bMGL·C_14_ (palmitoylglycerol mimic) and bMGL·C_16_ (stearoylglycerol mimic) ligand complexes were crystallized under the same condition equally yielding crystals of space group *P*2_1_. These structures were determined at resolutions of 1.85 and 2.2 Å, respectively. These crystals differed from the bMGL-C_12_ structure in that there were four molecules in the asymmetric unit with a different crystal packing arrangement. In all four independent molecules, electron densities for the ligands bound to active site residue Ser-97 were observed (Fig. 1, *D*, and *E*; PDB codes 4KE8 and 4KE9).

The bMGL(D196N) variant was crystallized in the presence of 1-LG (C_12:0_) and 1-OG (C_18:1_) yielding crystals of space group *P*2_1_2_1_2_1_ with six molecules in the asymmetric unit. The resolutions of the datasets were 2.8 and 1.7 Å, respectively. Of both crystals, we only interpreted the electron density of the bMGL(D196N)-1-LG crystal in chain A as substrate. The electron density for the glycerol moiety of 1-LG was well defined; however, the density for carbon atoms 7 and 8 of the fatty acid chain was not observed indicating flexibility (Fig. 1*F*; PDB code 4KE6). Attempts to co-crystallize bMGL(D196N) with 1-OG did not result in a complex structure. Instead, a crystal form of the free enzyme in space group *P*2_1_2_1_2_1_ was obtained containing six molecules in the asymmetric unit (PDB code 4KEA). The x-ray data collection and refinement statistics are listed in Tables 1–3.

### bMGL Shows a High Degree of Conformational Plasticity of the Cap

The first crystal structures of hMGL in its free form and in complex with different inhibitors revealed the cap in an open conformation (21, 22). In 2011, Schalk-Hihi *et al.* (23) determined the structure of hMGL in complex with a reversible, noncovalent inhibitor. This structure shows significant conformational changes in the cap region compared with the lipase in its free form and to hMGL in complex with the inhibitor SAR629 (21, 22). These changes resulted in a complete closure of the binding pocket of the lipase with concomitant electrostatic differences and led to a proposal that hMGL might dissociate from the membrane during the catalytic cycle (23). bMGL crystallized in its free form and in complex with PMSF also resulted in an open conformation, herein referred to as conformation I. MD simulations of bMGL showed that the plasticity of the cap can result in the closure of both the main binding pocket and the proposed glycerol exit hole (24). So far, very little is known about the link between these conformational changes and their impact on MGL activity.

**FIGURE 2.**
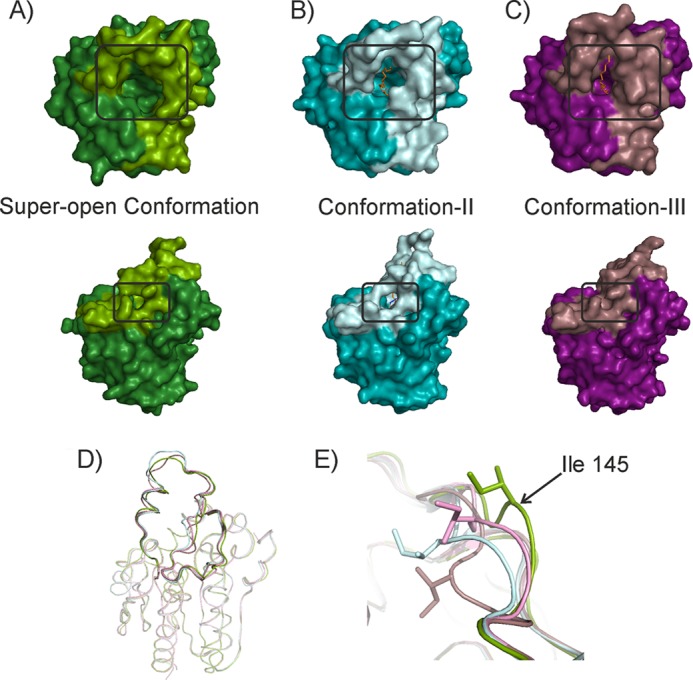
**Conformational plasticity of the cap.** *A–C*, surface representations of bMGL with the cap (Asn-120–Thr-164) and the core in different *color shades. A*, super-open conformation found in the free form of bMGL (D196N) chain A; *B*, conformation II; and *C*, conformation III. The main binding pocket and the glycerol exit hole are marked by *black boxes. D, ribbon* representation depicting the different cap conformations in bMGL. *E*, cap residue Ile-145 undergoes large conformational changes in the different conformations. Color coding for all panels is as follows: super-open conformation observed in chain A of free bMGL(D196N) is *green*; conformation II observed in the bMGL·C_14_ complex chain A is *cyan*; conformation III observed in the bMGL·C14 complex chain C is depicted in *violet*.

As the first remarkable feature of the different structures presented here, we highlight that bMGL captures different conformations of the cap region thus showing experimental proof of the predicted cap plasticity (Fig. 2); bMGL(D196N) co-crystallized with 1-OG did not yield a complex structure but a structure of free bMGL with six different chains in the asymmetric unit. Interestingly, the cap region samples open and partially restricted conformations in this structure, including a “super-open conformation” in chain A, with the access to the substrate binding pocket even more open than observed previously (Fig. 2*A*). Chains C–F adopt almost identical backbone conformations as observed in conformation I in the free form of bMGL and in the bMGL·PMSF complex (24). In these molecules the main substrate binding pocket and the proposed exit hole are both in an open conformation. Chain B adopts yet another conformation, showing a partially restricted binding pocket and the glycerol exit hole in an open conformation (referred to as conformation II). The bMGL·C_12_ complex structure shows the cap region in an open conformation similar to conformation I. Electron densities for residues Thr-133–Glu-137 in the cap region are absent in the C_12_ complex structure, similar to the free form of bMGL and the bMGL·PMSF complex (root mean square deviation of all Cα atoms, 0.13 and 0.14 Å, respectively) (24). The bMGL complex structures with the C_14_ and C_16_ ligands bound in the active site show clear electron densities for all residues of the cap region. Interestingly, chains A and B of the bMGL·C_14_ and bMGL·C_16_ ligand complexes show a partially restricted binding pocket and an open glycerol exit hole similar to conformation II (Fig. 2*B*). Chains C and D of the bMGL-C_14_, and bMGL-C_16_ ligand structures show even more pronounced restriction of access to the binding pocket accompanied by the closure of the glycerol exit hole denoted as conformation III (Fig. 2*C*).

The different conformations are neither caused by crystal contacts nor are they directly correlated to the specific ligands investigated here. Hence, these data suggest the cap movement in bMGL to be stochastic. One might speculate that flexibility in this region may be required for substrate and product entry and exit, respectively, and/or membrane interaction. The super-open conformation with the side chain of Ile-145 pointing away from the substrate binding pocket exposes the hydrophilic backbone of Ile-145 connecting the hydrophilic environment with the polar bottom of the binding pocket. Thus, the open conformation could facilitate the binding of the substrate. The significance of the cap movement with respect to the different catalytic steps is currently not known. It can be assumed that substrate binding requires an open conformation for steric reasons. The closed conformation provides a more hydrophobic environment potentially preventing substrate escape during catalysis. Yet it remains to be elucidated whether different catalytic steps such as the formation of the acyl-enzyme intermediate or the release of the different reaction products require different cap conformations.

#### 

##### Ile-145 Acts as Gatekeeper for the Substrate Entrance Tunnel and the Glycerol Exit Hole

**FIGURE 3.**
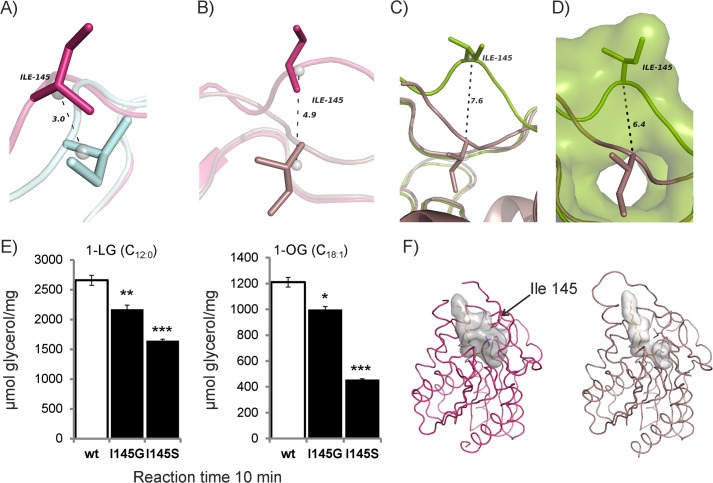
**Ile-145 acts as gatekeeper and restricts the size of the binding pocket.** *A*, close-up view highlighting the conformation of Ile-145 (*sticks*) and surrounding residues observed in conformation II of the bMGL C_14_ complex (*cyan*) and conformation I (*pink*). *Gray spheres* in *A–C* represent the geometric centers of the residues calculated by PyMOL. *B*, movements of Ile-145 (*sticks*) observed in conformation III (*dirty violet*) in comparison with conformation I (*pink*). *C*, comparison of Ile-145 in super-open conformation (*green*) and conformation III (*dirty violet*). *D*, close-up view and surface representation of super-open conformation (*green*) compared with conformation III (*dirty violet*). *E*, activity chart of wild-type bMGL and bMGL mutants I145G and I145S. *Bar diagram* depicting MG hydrolase activity toward 1-LG and 1-OG after a 10-min reaction time. All experiments were performed in triplicate and are representative of at least three experiments. Data are presented as mean ± S.D. Statistical significance was determined by Student's unpaired *t* test (two tailed) calculated in Excel (***, *p* < 0.001; **, *p* < 0.01; *, *p* < 0.05). *F*, binding cavity (*gray surface*) of the bMGL·C_12_ ligand complex (*pink ribbon*) in open conformation calculated by Casox (39). bMGL·C_14_ ligand complex (*dirty violet ribbon*) showing binding cavity (*gray surface*) in restricted conformation (conformation III). The ligands and residue Ile-145 are shown as *sticks*.

The different conformations observed provide an experimental corroboration for conformational plasticity of the bMGL cap region (Fig. 2). Therefore, we wanted to study the residues involved in mediating these conformational changes. A comparison between the super-open conformation (as observed in the free form of bMGL(D196N) chain A), conformation I (as observed in free bMGL, bMGL·PMSF complex, and the bMGL·C_12_ complex, and four molecules of free bMGL(D196N)), conformation II (as observed in two molecules of bMGL·C_14_ and bMGL·C_16_ complex structures and one molecule of free bMGL(D196N)), and conformation III (as observed in chains C and D of the bMGL·C_14_ and bMGL·C_16_ complex structures) shows that the position and conformation of Ile-145 differ extensively (Fig. 2, *D* and *E*). Inspection of Ile-145 in conformations I and II shows that the backbone Cα atom position of Ile-145 differs by about 2.7 Å accompanied by 1.8- and 5.1-Å movements of the CG2 and the CD1 side chain methyl groups, respectively. The geometric centers of Ile-145 differ by 3.0 Å (Fig. 3*A*). This results in a partly restricted conformation of the binding pocket in conformation II. Again, in conformation III the residue playing a crucial role in the observed changes is Ile-145. The CG2 and CD1 methyl groups are flipped 4.1 and 9.6 Å away from the open conformation, respectively. The geometric centers of Ile-145 differ by 4.9 Å (Fig. 3*B*). In the super-open conformation, the side chain of Ile-145 points into the solvent region without engaging in any crystal contacts (Fig. 3, *C* and *D*). The geometric center of Ile-145 in the super-open and conformation III differs by 7.6 Å (Fig. 3*C*). The closure of the exit hole in conformation III is mediated by a 6.4-Å movement of the main chain Cα of Ile-145 compared with the super-open conformation (Fig. 3*D*). In the bMGL·C_14_ complex, the movement of Ile-145 also restricts the orientation of the azide headgroup of the C_14_ ligand bound to Ser-97. In conformation III, Ile-145 engages in close contacts with the ligand, which results in positioning of the azide headgroup deep within the binding pocket to avoid steric clashes with Ile-145.

Thus, our crystal structures clearly show that Ile-145 plays a crucial role in engaging in conformational changes, modifying access to the substrate-binding site of bMGL, and determining open and closed states of the exit hole (Fig. 3). These observations pave the way for an intriguing set of questions relating to critical residues and the functional relevance of these different conformations. We therefore investigated whether this residue also influences the catalytic activity of the lipase. Two new variants of bMGL were generated by replacing Ile-145 with Ser and Gly, respectively. The variants were tested for MG hydrolase activity using 1-lauroyl-*rac*-glycerol (C_12:0_) and 1-oleoyl-*rac*-glycerol (C_18:1_). As reported before, WT-bMGL had a more than 2-fold higher activity toward the medium chain 1-LG compared with the longer chain 1-OG (25, 26). Compared with the wild-type protein, decreased activity levels were observed for the bMGL I145G variant against 1-OG and 1-LG (82% remaining activity for both substrates). Interestingly, the I145S variant harboring a polar side chain showed an even more drastic loss of activity toward theses substrates (38 and 62% for 1-OG and 1-LG, respectively) (Fig. 3*E*). This shows that increasing the flexibility of this loop region and concomitantly removing the hydrophobic side chain reduces activity. The introduction of a polar side chain has an even bigger effect indicating that a hydrophobic side chain is required at this position for optimal activity. One might speculate that such a hydrophobic residue aids in recruitment of the substrate, which itself harbors a long hydrophobic alkyl chain.

Next, we looked at the size of the substrate binding pocket in more detail. Analysis of the binding cavity revealed that the movement of Ile-145 from conformation I to conformation III squeezes the space within the binding pocket (39). Thus, it is tempting to speculate that conformation III (Fig. 3*F*) could limit the cavity to bind only MG and not diacylglycerol or triacylglycerol as bMGL shows no activity toward these substrates (26).

### Identification of the Binding Mode of the Substrate and Importance of Glu-156 for Glycerol Binding

**FIGURE 4.**
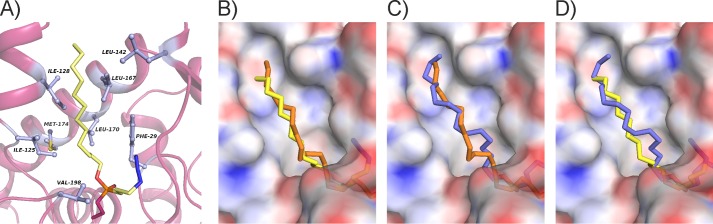
**Binding of the alkyl chain moiety in the substrate binding pocket.** *A*, hydrophobic interactions of bMGL with the alkyl chain of the ligand (key residues are shown as *light blue balls* and *stick representations*). *B–D*, surface colored according to electrostatic potential of bMGL in complex with C_16_ ligand. Electrostatics were calculated using APBS and visualized using the APBS plugin in PyMOL (46). *B*, comparison of bMGL covalently bound to the C_14_ ligand (*orange sticks*) superposed with C_12_ ligand (*yellow sticks*). *C*, comparison of bMGL covalently bound to C_14_ ligand (*orange sticks*) superposed with C_16_ ligand (*blue sticks*). *D*, comparison of bMGL covalently bound to C_16_ ligand (*blue sticks*) superposed with C_12_ ligand (*yellow sticks*).

**FIGURE 5.**
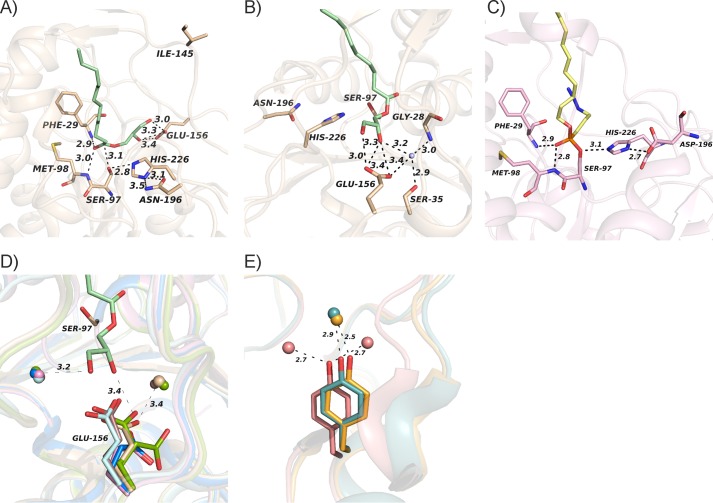
**Polar bottom of the substrate binding pocket.** *A*, close-up view of 1-LG (g*reen sticks*) in the active site of bMGL (*wheat*). *B*, hydrogen bonding network (*dashes*) of a water molecule (*sphere*) and bMGL residues (*wheat sticks*) with the glycerol moiety. The catalytic residues and the residues interacting with the glycerol moiety are shown as *sticks. C*, bMGL (*pink*) with the C_12_ substrate analog (*yellow sticks*) covalently bound to the active site serine. Residues of the catalytic triad and residues forming the oxyanion hole are shown as *sticks. D*, close-up view of the superposition of the active site of bMGL in free form (*blue*), in complex with C_12_ ligand (*pink*), C_14_ ligand (*dirty violet*), C_16_ ligand (*cyan*), and 1-LG (*wheat*) and the free form of the bMGL(D196N) variant (*green*) showing conserved water molecules (*spheres* in respective colors) stabilizing the glycerol moiety. *E*, zoomed in view of Tyr-194 in the hMGL structures in free form (PDB code 3HJU, *cyan*; PDB code 3JW8, *orange*), in complex with a noncovalent inhibitor (PDB code 3PE6, *salmon*) showing conserved water molecules (*spheres*) potentially stabilizing glycerol binding. Tyr-194 is represented as *sticks*.

The bMGL·C_12_, bMGL·C_14_, and bMGL·C_16_ complex structures show that the hydrophobic nature of the substrate binding pocket plays a major role in stabilizing the fatty acid moiety of the substrate. Representative of these contacts, the side chains of residues from both the α/β-hydrolase core and the cap region, including Phe-29, Ile-125, Ile-128, Leu-142, Leu-167, Leu-170, Met-174, and Val-198, form hydrophobic contacts with the alkyl chain of the C_12_-ligand bound at the active site in the bMGL-C_12_ complex (Fig. 4*A*). The otherwise flexible fatty acid chain of an MG substrate is observed in a single conformation in the crystal structure. The backbone NH groups of Phe-29 and Met-98 form hydrogen bonds with the O2 oxygen atom of the phosphonate group of the C_12_ ligand, highlighting that these residues form the oxyanion hole during the course of MG hydrolysis (Figs. 4*A* and 5*C*). In the free form of the lipase, a water molecule occupies this position (24).

bMGL has been reported to have a preference for C_8_–C_14_ chains with 1-LG (C_12_) identified as the best substrate among those tested. Our results are in agreement with these previous reports (Fig. 3*E*) (25, 26). The structures reported here reveal the impact of chain length on the interactions of substrate with the binding site. The bMGL·C_12_ ligand complex shows the aliphatic chain of the ligand in an almost linear conformation protruding toward the surface (Figs. 4*B* and 1*C*). In the bMGL·C_14_ complex, a bend in the aliphatic chain allows the longer chain to be accommodated in the binding pocket (Figs. 4*C* and 1*D*). The terminal carbon atoms of both the C_14_ and C_16_ alkyl chains are located at approximately the same distance from the surface of the protein, close to residues that line the entrance of the binding pocket (Fig. 4*C*). The C_16_ ligand is also fully accommodated in the binding pocket, despite the four additional CH_2_ groups, due to alkyl chain bending (Figs. 4*D* and 1*E*). The necessity for bending might explain the lower substrate turnover rate of bMGL with respect to MGs with a longer fatty acid chain.

Next, we analyzed the binding mode of the glycerol moiety of the MG substrate. To stall the catalytic activity, yet retain a WT-like architecture of the catalytic center, we generated an inactive variant of bMGL by introducing an D196N point mutation. The structure of the bMGL(D196N)·1-LG complex shows the interactions between the glycerol moiety of an MG substrate and residues at the bottom of the substrate binding pocket. The carbonyl carbon of 1-LG is located 3.1 Å away from the oxygen OG of the nucleophilic Ser-97 (Fig. 5*A*). The glycerol moiety of 1-LG forms contacts with the side chain of Glu-156 and a water molecule. The hydroxyl group of Ser-35 and the backbone nitrogen of Gly-28 are also engaged in interactions with the glycerol moiety via this water molecule (Fig. 5*B*).

Interestingly, two water molecules are positionally conserved in the other bMGL structures (Fig. 5*D*). The fact that only one water was observed in the bMGL(D196N)·1-LG complex structure might be due to the lower resolution of that dataset.

### bMGL Complexes Provide Detailed Insights into the First Steps of the Lipolytic Reaction

The catalytic mechanism of bMGL is expected to follow the typical hydrolysis mechanism of α/β-hydrolases involving the catalytic triad residues Ser-97, Asp-196, and His-226 and the oxyanion hole forming residues Met-98 and Phe-29 (Fig. 5*A*) (24, 40). Asp-196 forms a hydrogen bond to a histidine imidazole nitrogen thus enabling His-226 to act as the general base and to accept the proton from the catalytic serine. The deprotonated serine acts as a powerful nucleophile attacking the carbonyl carbon of the MG substrate. The spatial arrangement of the catalytic triad residues in the free enzyme structure is consistent with this general reaction scheme (24). In the D196N mutant, in addition to a loss of charge, the tautomeric state of the histidine side chain is likely to be altered. The reaction is stalled before the nucleophilic attack with the entire ligand in the active site representing the Michaelis complex. The distance between Ser-OG and the carbonyl carbon of the 1-LG substrate is 3.1 Å (Fig. 5*A*). In analogy to serine proteases, the main chain atoms of Met-98 and Phe-29 form the oxyanion hole, which would stabilize the tetrahedral intermediate formed in the course of MG hydrolysis. Accordingly, the main chain NH groups are in hydrogen bonding distance from the 1-LG carbonyl-oxygen in the structure (Fig. 5*A*).

We synthesized *p-*nitrophenol esters of alkyl phosphonic acids and used them as substrate analogs with different alkyl chain lengths. These compounds react irreversibly with the nucleophilic serine of α/β-hydrolases and are used frequently to capture the tetrahedral reaction intermediates of lipases (41–45). Consequently, the inhibitor complexes go one step further along the reaction trajectory compared with the D196N 1-LG complex; Ser-97 exerted its nucleophilic attack on the phosphorus-atom of the MG-mimicking ligand, and then the reaction is stalled. The *p-*nitrophenol moiety is released and no longer present in the active site (Fig. 5*C*). The irreversibly and covalently bound ligand is captured in the bMGL·C_12_, bMGL·C_14_, and bMGL·C_16_ complex structures (distance between Ser-OG and the phosphorus atom, 1.6 Å) mimicking the tetrahedral intermediate of carboxylic acid ester hydrolysis. The ligands also form hydrogen bonds to the oxyanion hole residues Met-98 and Phe-29. The distances between backbone hydrogen donor nitrogens and phosphonate oxygens are 2.8 and 2.9 Å (Fig. 5*C*). The next steps in the catalytic reaction of an MGL substrate would be protonation and release of the glycerol moiety. His-226 is very likely to act as the proton donor. Following glycerol release, the ester carbonyl would likely be held in place by Met-98 and Phe-29 in the acyl-enzyme intermediate. In the free state of bMGL, a further water molecule is located in hydrogen bonding distance from His-226 NE2 that could occupy a similar position to a water molecule required for fatty acid release. The position and orientation of the ligands in our structures are consistent with such a prototypical hydrolysis mechanism.

### Conservation of Structural Features of bMGL in Human MGL

The structure of bMGL in its free form revealed structural conservation of the α/β-hydrolase core region and unexpected evolutionary conservation of the cap architecture between human and bacterial proteins (24). The structures of bMGL in complex with substrate and the analogs reported here shed light on the conservation of additional structural features important for the hydrolytic action of these two proteins.

#### 

##### Binding Pocket Hydrophobicity

The lipophilic potential of bMGL calculated using VASCo indicated that the hydrophobicity of the protein is largely restricted to the main substrate binding pocket (24, 39). The structures of bMGL in complex with 1-LG, C_12,_ C_14_, and C_16_ ligands corroborate the importance of the hydrophobicity of the binding pocket in the otherwise polar and water-soluble protein (Fig. 4). The hydrophobic interactions between the side chains of the residues lining the pocket and the aliphatic carbon chain of the ligand provide an ideal environment for the binding of the fatty acid moiety of an MG. The structures of hMGL and bMGL show that the hydrophobicity of the binding pocket is conserved between the two lipases (21–23). It suggests that despite the low sequence identity of only 17%, the conservation of the hydrophobicity of the substrate binding pocket is one of the essential factors for MGL function.

##### Glycerol Binding

Although structures of hMGL have been determined in free form and in complex with inhibitors, very little is known about the glycerol binding pocket of MGLs. The structure of bMGL in complex with 1-LG provides first insights into the important role of Glu-156 and a water molecule for binding the glycerol moiety of the substrate. Our bMGL structures determined in free form and in complex with ligand show that one water molecule is directly involved in binding the glycerol moiety, but two are positionally conserved in all the other bMGL structures (Fig. 5, *B* and *D*). In the human ortholog, docking studies indicated that Tyr-194 interacts with the glycerol moiety. This represents a difference to bMGL where we identified Glu-156 as a key residue for glycerol binding (22). hMGL also harbors conserved water molecules in the glycerol binding pocket. In the structure of hMGL in its free form (PDB code 3HJU (21)), Tyr-194 forms a hydrogen bond with a water molecule located at a distance of 2.7 Å (Fig. 5*E*) (22). In another structure (PDB code 3JW8), a water molecule in a similar position forms a hydrogen bond with this tyrosine residue at a distance of 2.5 Å. In the hMGL structure in the closed conformation, two water molecules engage in hydrogen bonding interactions with Tyr-194 (PDB code 3PE6 (23)). Interestingly, these water molecules are located at different positions compared with the other two hMGL structures. This could be due to the fact that the ligand co-crystallized with the protein extends into the glycerol binding pocket. Hence, water molecules probably play a role in glycerol binding in human as well as bacterial MGLs.

##### Isoleucine Residues Undergo Conformational Changes in the Cap

**FIGURE 6.**
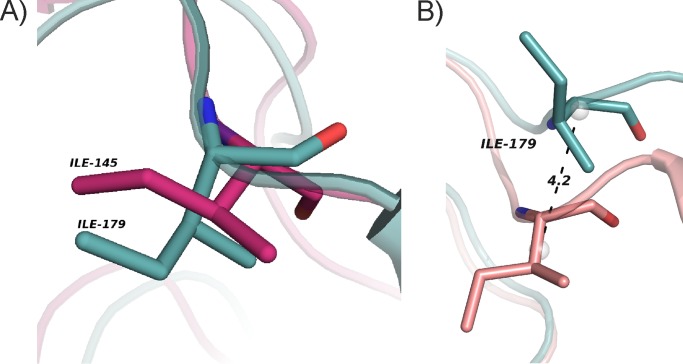
**Conservation of structural features.** *A*, structural superposition of hMGL (PDB code 3HJU; *cyan*) with bMGL in free form (PDB code 3RM3, *pink*) showing positional conservation of Ile-179 and Ile-145, respectively. *B*, superposition of hMGL in open (PDB code 3HJU; *cyan*) and closed (PDB code 3PE6, *salmon*) conformation, highlighting the movement of Ile-179 (*sticks*). *Gray spheres* represent the geometric center of the residues calculated by PyMOL.

bMGL structures reveal that Ile-145 is the major residue undergoing conformational changes in the cap region acting as a gatekeeper. These movements affect the access to the substrate binding pocket and the closure of the exit hole. The structure of hMGL determined in the closed conformation with a noncovalent inhibitor possesses strikingly similar properties. The closure of the exit hole in this complex coincides with movements of Ile-179 (23). This residue is located at an equivalent position to Ile-145 in bMGL with the backbone atoms lining the glycerol exit hole (Fig. 6*A*). Analysis of the three-dimensional structure of hMGL reveals that both the side chain and the main chain of Ile-179 change conformation to open and close the proposed glycerol exit hole (Fig. 6*B*).

In summary, the structures of bMGL in complex with substrate and substrate analogs reveal the mode of substrate binding in MGLs and capture different stages of the lipolytic reaction. In complex structures with substrate analogs of different chain lengths, bending of longer alkyl chains is observed, which might contribute to faster turnover rates of MG substrates with short chains. The bMGL structures shed light on the stochastic equilibrium between open and restricted cap conformations. Analysis of the binding pocket leads to the hypothesis that restricted conformations result in substrate selectivity toward MG only. It remains to be seen whether these cap conformations are essential for catalysis and release of reaction products. These questions might be addressed in future studies by using a combined approach of time-dependent soak-freeze x-ray crystallography, site-directed spin labeling, and NMR spectroscopy.

## Footnotes

4 The abbreviations used are:

MGL: monoacylglycerol lipase
1-LG: 1-(*rac*)-lauroylglycerol
PDB: Protein Data Bank
bMGL: bacterial MGL
hMGL: human MGL
1-OG: 1-oleoyl-*rac*-glycerol
MG: monoacylglycerol.
